# The prevalence of inherited metabolic disorders in Estonian population over 30 years: A significant increase during study period

**DOI:** 10.1002/jmd2.12325

**Published:** 2022-08-24

**Authors:** Elis Tiivoja, Karit Reinson, Kai Muru, Kristi Rähn, Kristina Muhu, Laura Mauring, Tiina Kahre, Sander Pajusalu, Katrin Õunap

**Affiliations:** ^1^ Department of Clinical Genetics, Institute of Clinical Medicine University of Tartu Tartu Estonia; ^2^ Department of Clinical Genetics, Genetic and Personalized Medicine Clinic Tartu University Hospital Tartu Estonia; ^3^ Eye Clinic Tartu University Hospital Tartu Estonia; ^4^ Department of Laboratory Genetics, Genetic and Personalized Medicine Clinic Tartu University Hospital Tartu Estonia

**Keywords:** diagnostic efficacy, epidemiology, exome sequencing, inborn errors of metabolism, inherited metabolic disorders, prevalence

## Abstract

Inherited metabolic disorders (IMD) are a group of hereditary diseases wherein the impairment of a biochemical pathway is intrinsic to the pathophysiology of the disease. Estonia's small population and nationwide digitalised healthcare system make it possible to perform an epidemiological study that covers the whole population. A study was performed in Tartu University Hospital, which is the only tertiary care unit in Estonia for diagnosing patients with IMD, to define the prevalence and live birth prevalence of IMDs and the effectiveness of new diagnostic methods on the diagnosis of IMD. During the retrospective study period from 1990 to 2017, 333 patients were diagnosed with IMD. Statistical analysis showed a significant increase in IMD diagnoses per year from 0.47 to 2.51 cases per 100 000 persons (*p* < 0.0001) during the study period. Live birth prevalence of IMD in Estonia was calculated to be 41.52 cases per 100 000 live births. The most frequently diagnosed IMD groups were disorders of amino acid metabolism, disorders of complex molecule degradation, mitochondrial disorders, and disorders of tetrapyrrole metabolism. Phenylketonuria was the most frequently diagnosed disorder of all IMD (21.6%). Our results correlated well with data from other developed countries and, along with high birth prevalence, add confidence in the effectiveness of our diagnostic yield. Implementation of new diagnostic methods during study period may largely account for the significant increase in the number of IMD diagnoses per year. We conclude that the implementation of new diagnostic methods continues to be important and contributes to better diagnosis of rare diseases.

**Synopsis**

Statistical analysis showed a significant increase in inherited metabolic disorders diagnoses per year from 0.47 to 2.51 cases per 100 000 persons (*p* < 0.0001) during 1990–2017 in Estonia, which shows that the implementation of new diagnostic methods contributes to better diagnosis of rare diseases.

## 1 INTRODUCTION

Inherited metabolic disorders (IMD) are a group of hereditary diseases wherein the impairment of a biochemical pathway is intrinsic to the pathophysiology of the disease.^1^ Though individually rare, the estimated birth prevalence of all IMD combined is 50.9 per 100 000 live births (LB).^2^ Presently, over 1450 such disorders have been described.^3^

IMD were first recognized by Sir Archibald Garrod at the beginning of 20th century, based on his studies on alkaptonuria (OMIM: #203500).^4^ Newborn screening (NBS) started with phenylketonuria (PKU) (OMIM: #261600) in the 1960s^5^ and expanded with the introduction of tandem mass spectrometry in the 1990s.^6^ In the 21st century, the world has seen huge advancements in the field of genetics. The development of various “‐omics” technologies to sequence exomes (exome sequencing; ES) and genomes has permitted the discovery of new diseases, which has significantly increased the detection and diagnosis of new IMD.^7^

The first sub‐classification of IMD dates back to 1960, when 10 groups of disorders were defined (Stanbury).^8^ Since then, many additional classifications have been proposed, the most recent being the International Classification of Inherited Metabolic Disorders (ICIMD), published in 2021,^3^ which was used in the current study to assign the disorders into groups. Estonia is a small Northern European country with a total population of 1 319 133 people, of which 275 399 are 0–19 years old (data from Statistics Estonia on January 1, 2018).^9^ The Genetics and Personalized Medicine Clinic of Tartu University Hospital (GPMC TUH) is the only tertiary care unit in Estonia for the diagnosis and care of patients with IMD. This clinic has locations in two cities – Tallinn, for Northern Estonia, and Tartu, for Southern Estonia. The modest size of the country and its nationwide digitalized healthcare system make it feasible to perform an epidemiological study that covers the whole population and maximizes the number of patients detected with IMD. The GPMC TUH is also a member of the European Reference Network for Hereditary Metabolic Diseases (Metab ERN) since 1 January, 2022, and obtained an online accreditation in 2021.

The aims of this study were to retrospectively identify the nationwide prevalence of all diagnosed IMD in Estonia, to find the LB prevalence of these disorders from 1980 to 2017, and to compare these results with previously published data. Here, we will also describe the implementation of new diagnostic methods in Estonia over these years and their impact on the diagnosis of different IMDs.

## 2 MATERIALS AND METHODS

The study was approved by Research Ethics Committee of the University of Tartu (278/T‐19 on 19.02.2018 and 288/M‐17 on 17.12.2018) and was conducted in accordance with the rules of Declaration of Helsinki.^10^

The study was performed at the GPMC TUH, which is the only center in Estonia that focuses on molecular diagnostics and genetic counseling, including for patients diagnosed with IMD. The presence of a branch of our center in the country's capital, Tallinn, facilitates the transfer of our data to the national level. Data were retrospectively gathered from the archives of the GPMC TUH, from the hospital's electronic database, and from the molecular genetics laboratory. We collected the data of all patients who received the diagnosis of IMD from 1990 to 2017. Of the final database, 113 patients that were born before 1990, but received a diagnosis in 1990 or later, were included in our study group. Electronic records were available since the year 2008. Data from other clinics outside the GPMC TUH were obtained only from the electronic database from years 2008 to 2017. The analysis included both primary conditions and comorbidities encoded with ICD‐10 (the 10th revision of the International Classification of Diseases) codes E70–E72, E74–E77, E79–E80, E83, E88 and G31. G31 has been used in some cases to code neurodegenerative IMDs, like mitochondrial myopathy. The limitation of this study is that many IMDs included in the current ICIMD are not associated with any ICD‐10 code. We have usually used E88.8 code for other specified IMD.

We initially gathered 2997 records, and from these, we selected for analysis only those cases in which IMD was diagnosed or confirmed. All entries were examined one‐by‐one, to include only patients with a correctly diagnosed IMD. For each patient, parameters including: personal identification code, year of birth, diagnosis, year of diagnosis, results of biochemical, enzymatic and molecular genetics analyses, treatment and present state were documented.

Inclusion criteria were applied as follows:

- Molecular and/or enzymatic confirmation of the clinically and/or biochemically suspected IMD;
- Symptomatic disease with characteristic biochemical changes only. These cases were diagnosed before molecular diagnostics was widely available (e.g., PKU);
- Symptomatic disease with characteristic biochemical changes when the subject's first degree relative had a molecularly or enzymatically confirmed diagnosis (e.g., porphyria);
- Specialist's confirmation of the diagnosis based on an objective examination of the patient was used only in the case of ocular‐ (OMIM: #300650) and oculocutaneous albinism (OMIM: #203100).

Hemochromatosis (OMIM: #235200) (E83.1) was excluded in the early stage of data collection because in many individuals, there was no molecular confirmation of disease, or because the clinical and/or biochemical status was unknown. After re‐evaluation of all data and the exclusion of unsuitable entries, the final database for analysis included 333 patients with IMD. ICIMD was used for categorizing the disorders into groups.^3^

## 3 STATISTICAL METHODS

### 3.1 Calculation of prevalence of diagnosed IMD

The prevalence of IMD was defined as the total number of patients with IMD diagnosed during the period from 1990–2017, divided by the number of people living in Estonia within the same period. The prevalence of IMD was estimated using a general linear model (GLM) analysis using R version 4.0.2 (Team).^11^ A Poisson distribution was assumed for the prevalence cases, and the default logarithmic link function was used. The only variable in the model was the observation year. The mean (expected) prevalence rate for a given year and the corresponding 95% confidence limits were calculated using R. Differences were considered statistically significant if the *p*‐value was less than 0.05.

### 3.2 Calculation of live birth prevalence

The LB prevalence of IMD was calculated by dividing the total number of patients with IMD born during the period from 1980 to 2017 by the recorded number of LB for the same period. The 95% confidence interval was calculated based on the Poisson distribution.^12^ According to the database of Statistics Estonia, there were 647 869 LB between the years 1980 and 2017, inclusive.^9^

## 4 RESULTS

### 4.1 The prevalence of diagnosed IMD

**FIGURE 1:**
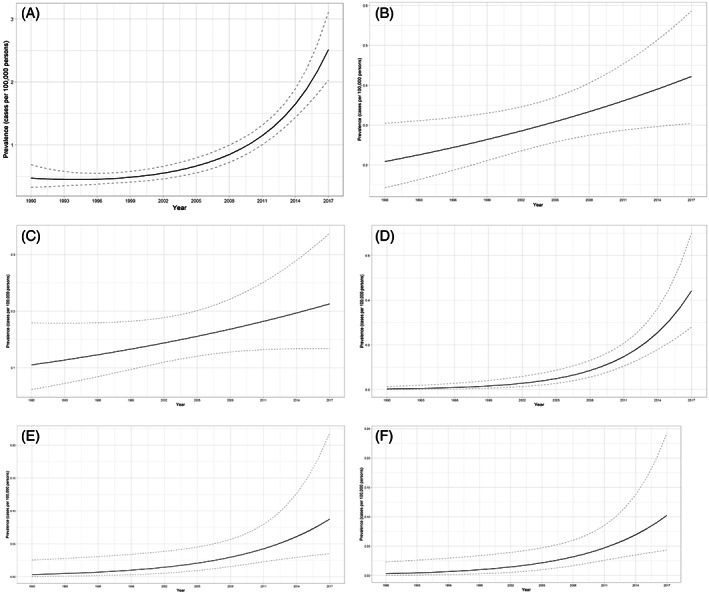
Numbers of inherited metabolic disorder (IMD) diagnoses per year per 100 000 persons in Estonia during the period from 1990–2017. (A) All IMD; (B) disorders of amino acid metabolism; (C) disorders of complex molecule degradation; (D) mitochondrial disorders; (E) disorders of energy substrate metabolism; (F) disorders of fatty acid and ketone body metabolism

During the period from 1990 to 2017, 333 patients were diagnosed with IMD. Statistical analysis showed an increase in the number of IMD diagnoses per year, from 0.47 (95% CI 0.33–0.69) cases per 100 000 persons to 2.51 cases (95% CI 2.03–3.11) per 100 000 persons (Figure 1A, Table 1). This increase was statistically significant (*p* < 0.0001).

**TABLE 1:** The prevalence of diagnosed IMD during the period from 1990 to 2017

IMD	Prevalence in 1990 per 100 000 persons	Prevalence in 2017 per 100 000 persons	*p* value
All IMD	0.47 (95% CI 0.33–0.69)	2.51 (95% CI 2.03–3.11)	*p* < 0.0001^a^
Disorders of amino acid metabolism	0.20874 (95% CI 0.14296–0.30479)	0.42221 (95% CI 0.30413–0.58615)	*p* = 0.02267^a^
Disorders of complex molecule degradation	0.10523 (95% CI 0.06174–0.17932)	0.21297 (95% CI 0.13419–0.33802)	*p* = 0.105
Mitochondrial disorders	0.00318 (95% CI 0.00073–0.01383)	0.44242 (95% CI 0.27941–0.70054)	*p* < 0.00001^a^
Disorders of energy substrate metabolism	0.00339 (95% CI 0.00045–0.0254)	0.08767 (0.03517–0.21856)	*p* = 0.01375^a^
Disorders of fatty acid and ketone body metabolism	0.0031 (95% CI 0.0004–0.0232)	0.1025 (95% CI 0.0433–0.2425)	*p* = 0.00716^a^

Abbreviation: IMD, inherited metabolic disorders.

^a^ Statistically significant.

The most frequently diagnosed disorder groups were disorders of amino acid metabolism (117 patients; 35.1%), disorders of complex molecule degradation (59 patients; 17.7%), mitochondrial disorders (35 patients; 10.5%) and disorders of tetrapyrrole metabolism (30 patients; 9.0%). All disorders of tetrapyrrole metabolism were different types of porphyrias.

Out of the 117 patients diagnosed with an amino acid metabolism disorder, 72 (61.5%) were diagnosed with PKU. Statistical analysis showed an increase in the number of diagnoses of disorders of amino acid metabolism from 0.20874 (95% CI 0.14296–0.30479) cases per 100 000 persons in year 1990 to 0.42221 (95% CI 0.30413–0.58615) cases per 100 000 persons in year 2017 (Figure 1B, Table 1). This increase was statistically significant (*p* = 0.02267).

Mucopolysaccharidoses (MPS) were the most frequent disorders of complex molecule degradation (20 out of 59 patients; 33.9%). The most common MPS subtypes were type II (OMIM: #309900) and type IIIA (OMIM: #252900), each diagnosed in eight patients (40% of total MPS diagnosed, respectively). Type VI (OMIM: #253200) was diagnosed in two patients (10%) and type I (OMIM: #252800) and VII (OMIM: #253220) were each diagnosed in one patient (5%). There was no statistically significant increase in the number of diagnoses of disorders of complex molecule degradation per year during the study period (*p* = 0.105), as the cases increased from 0.10523 (95% CI 0.06174–0.17932) cases per 100 000 persons in 1990 to 0.21297 (95% CI 0.13419–0.33802) cases per 100 000 persons in 2017 (Figure 1C, Table 1).

Our group of mitochondrial disorders includes mtDNA related disorders, disorders of mitochondrial gene expression, nuclear‐encoded disorders of oxidative phosphorylation, and other disorders of mitochondrial function. Statistical analysis showed an increase in the number of annual diagnoses of this group from 0.00318 (95% CI 0.00073–0.01383) cases per 100 000 person in 1990 to 0.44242 (95% CI 0.27941–0.70054) cases per 100 000 persons in 2017 (Figure 1D, Table 1). This increase was statistically significant (*p* < 0.00001).

There was also a significant increase in the number of diagnoses of disorders of energy substrate metabolism (*p* = 0.01375) from 0.00339 (95% CI 0.00045–0.0254) to 0.08767 (0.03517–0.21856) cases per 100 000 persons per year (Figure 1E) and in the number of diagnoses of disorders of fatty acid and ketone body metabolism (p = 0.00716) from 0.0031 (95% CI 0.0004–0.0232) to 0.1025 (95% CI 0.0433–0.2425) cases per 100 000 persons per year (Figure 1F, Table 1).

### 4.2 Live birth prevalence of IMD

**FIGURE 2:**
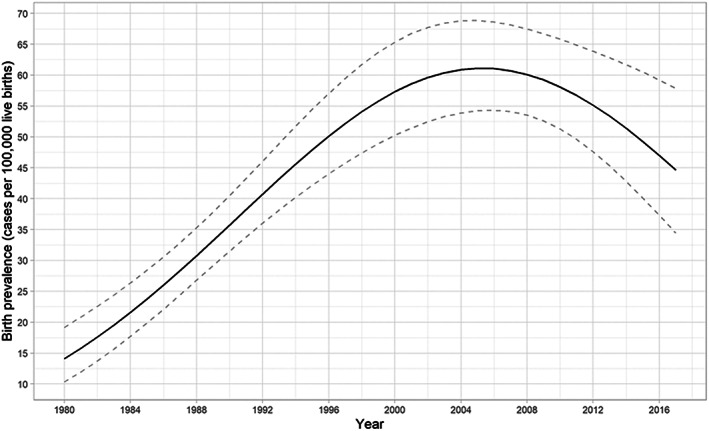
Live birth prevalence of inherited metabolic disorders in Estonia per 100 000 live births during the period from 1980 to 2017

Live birth prevalence of IMD in Estonia was calculated to be 41.52 (95% CI 37.45–45.96) cases per 100 000 LB (Figure 2). Different disorders, disorder groups, and their LB prevalence are shown in Table 2. The LB prevalence of disorders of amino acid metabolism, disorders of complex molecule degradation, and mitochondrial disorders were 16.82 (95% CI 14.27–19.75), 7.56 (95% CI 5.89–9.62) and 3.24 (95% CI 2.18–4.69) cases per 100 000 LB, respectively.

**TABLE 2:** Live birth prevalence of inherited metabolic disorders in Estonia, during the period from 1990 to 2017

IMD	ICIMD codes^3^	LB prevalence (per 100 000 LB)	95% CI
All IMD		41.52	37.45–45.96
Disorders of amino acid metabolism	IEM0056 ‐ IEM0186	16.82	14.27–19.75
Phenylketonuria	IEM0082	10.96	8.92–13.37
Disorders of complex molecule degradation	IEM0689; IEM0811 ‐ IEM0877	7.56	5.89–9.62
Mucopolysaccharidoses	IEM0858 ‐ IEM0868	2.93	1.93–4.32
Mitochondrial disorders	IEM0413 ‐ IEM0626	3.24	2.18–4.69
Disorders of energy substrate metabolism	IEM0389 ‐ IEM0403	1.54	0.85–2.64
Disorders of fatty acid and ketone body metabolism	IEM0627 ‐ IEM0645	1.39	0.73–2.45
MCAD deficiency	IEM0633	0.15	0.01–0.76
LCHAD deficiency	IEM0636	0.77	0.31–1.65
Congenital disorders of glycosylation	IEM0908 ‐ IEM1031	1.39	0.73–2.45

Abbreviations: CI, confidence interval; IMD, inherited metabolic disorders; LB, live birth; LCHAD, long‐chain L‐3 hydroxyacyl‐CoA dehydrogenase deficiency; MCAD, medium‐chain acyl‐CoA dehydrogenase deficiency.

PKU was the most frequently diagnosed disorder of all IMD, accounting for 21.6% of total IMD diagnosed, with a LB prevalence of 10.96 (95% CI 8.92–13.37) cases per 100 000 LB. Other frequently diagnosed disorders were porphyrias (30 cases; 9% of IMD diagnosed), MPS (20; 6%), ocular and/or oculocutaneous albinism (24; 7.2%) and Wilson's disease (OMIM: #277900) (18; 5.4%). The LB prevalence for MPS was 2.93 (95% CI 1.93–4.32) cases per 100 000 LB.

The LB prevalence of disorders of fatty acid and ketone body metabolism was 1.39 (95% CI 0.73–2.45) cases per 100 000 LB. The most common disorder of that group was long‐chain L‐3 hydroxyacyl‐CoA dehydrogenase (LCHAD) deficiency (OMIM: #609016), accounting for 45% of the diagnoses. The LB prevalence for LCHAD was 0.77 (95% CI 0.31–1.65) cases per 100 000 LB, and for medium‐chain acyl‐CoA dehydrogenase (MCAD) deficiency (OMIM: #201450), the prevalence was 0.15 (95% CI 0.01–0.76) cases per 100 000 LB.

The LB prevalence of congenital disorders of glycosylation (CDG) was 1.39 (95% CI 0.73–2.45) cases per 100 000 LB, the most common being PMM2‐CDG (OMIM: #601785), which constituted 67% of the CDG diagnoses.

The LB prevalence of disorders of energy substrate metabolism was 1.54 (95% CI 0.85–2.64) cases per 100 000 LB. X‐linked creatine transporter deficiency (OMIM: #300036) accounted for 70% of the diagnoses.

Cases diagnosed in clinics outside of the GPMC TUH accounted for 11.1% of all diagnosed cases, and 17.9% of the cases diagnosed from 2008–2017, inclusive. The diagnoses from other clinics were mainly porphyrias (disorders of tetrapyrrole metabolism) and oculocutaneous and ocular albinism (disorders of amino acid metabolism).

### 4.3 Advances in diagnostic methods of IEM

**FIGURE 3:**
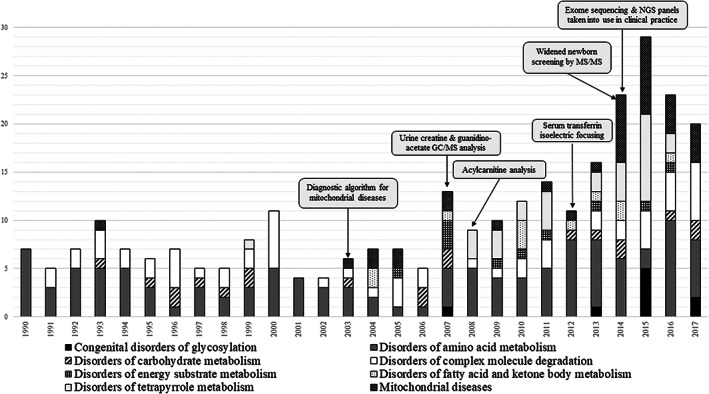
Diagnosed cases of different inherited metabolic disorder groups during the period from 1990 to 2017 in Estonia and the diagnostic methods implemented during the study period. For each new diagnostic method, the arrow points to the year in which it was implemented

Different diagnostic methods were implemented during study period in Estonia. The diagnostic algoritm for mitochondrial disorders in 2003,^13^ urinary creatine and guanidinoacetate gas‐chromatography/mass spectrometry (GC/MS) analysis in 2007,^14^ serum acylcarnitine analysis in 2008,^15^ serum transferrine isoelectric focusing (TIEF) in 2012,^16^ expanded newborn screening by tandem mass‐spectrometry (MS/MS) analysis in 2014,^17^ and next generation sequencing (NGS) panels and ES were taken into clinical use in 2014.^18^ The implementation of all those methods are summarized in Figure 3.

## 5 DISCUSSION

Here, we summarize our retrospective study of IMD diagnoses in Estonia for the years 1990–2017. Statistical analysis showed a significant increase in the number of IMD diagnoses per year in the study period, which we first attribute to the implementation of new diagnostic methods during study period. However, we cannot underestimate the consistent education and training of Estonian physicians along the care pathway, and finally the assembled organization of genetic service for diagnostics of IEM under Tartu University Hospital. As Estonia is a small‐digitalized country, the overall organization of the care pathway for IMDs is very smoothly organized. The number of diagnosed cases of selected disorder groups, along with the diagnostic methods implemented during the study period, are summarized in Figure 3.

The first diagnostic algorithm for mitochondrial disorders in Estonia was implemented in 2003.^13^ Statistical analysis showed a significant increase of diagnosed mitochondrial disorders during the study period from 1990 to 2017. This result suggests that the use of the algorithm may have contributed to improved diagnosis of mitochondrial disorders. In 2014, ES analysis was implemented in everyday clinical practice, nationwide. One study using ES in patients with an unsolved, but suspected mitochondrial disorder, showed a diagnostic yield of 57%; however, mitochondrial disorder was diagnosed only in 14% of patients.^19^

Urine creatine and guanidinoacetate GC/MS analysis was put into use in 2007 for the diagnosis of brain creatine deficiency syndromes, including creatine transporter defects caused by hemizygous *SCL6A8* gene variants. We have previously reported that the *SLC6A8* gene defect causes approximately 2% of X‐linked intellectual disability cases.^14^ No cases of X‐linked creatine transporter deficiency had been diagnosed in Estonia before the year 2007, and our study found a significant increase of diagnoses of disorders of energy substrate metabolism during the study period, suggesting this as another example of how implementation of a single biochemical method can lead to a significant improvement in diagnosis. Similarly, acylcarnitine analysis using MS/MS analysis was implemented in 2008,^13^ which is necessary for the biochemical diagnosis of disorders of fatty acid and ketone body metabolism. Statistical analysis in our retrospective study showed a significant increase in the diagnosis of this disorder group, as well, which we attribute to the impact of this method.

Serum TIEF analysis has been performed for screening CDG in Estonia since 2012.^16^ To underscore the critical diagnostic impact this had for CDG, in the years 2012–2017, eight patients were diagnosed with CDG, while before the year 2012, only one patient had received a diagnosis of a CDG, with the help of European expert center (Leuven). The prevalence of CDG in Europe is estimated to be 0.1–0.5 cases per 100 000 persons,^20^ while based on allele frequencies in the Genome Aggregation Database (gnomAD), the actual prevalence is estimated to be around 2 cases per 100 000 in Estonians.^21^ In our study, the LB prevalence based on diagnosed cases was detected as 1.39 cases per 100 000 LB. It shows that the implementation of TIEF analysis in routine clinical practice has given positive effect on detection of new CDG patients.

Screening for PKU in Estonia began in 1993.^22^ In 2014, an expanded newborn screening program was introduced, which added 18 new IMDs to the national NBS program.^23^ Among aminoacidopathies, we previously mainly diagnosed PKU cases, which is the most common IMD in Estonia, as in many other countries. However, since the introduction of the expanded screening program, we have diagnosed a wider range of aminoacidopathies, such as glutaric aciduria type 1 (OMIM: #231670), maple syrup urine disease (OMIM: #248600) and homocystinuria (OMIM: #236200). The statistical analysis in our retrospective study showed a significant increase in diagnoses of amino acid metabolism disorders during the study period. This may be a result of not only the expansion of the screening program (53% of diagnoses), but also, the introduction of genome‐wide diagnostic methods (6% of diagnoses). However, this result may also appear inflated by the inclusion of data from other departments, which became available in 2008. These data from other clinics included primarily diagnoses of oculocutaneous and ocular albinism, and porphyrias, and therefore, do not likely have a major effect on the interpretation of apparent diagnostic gains for other disorder groups.

ES‐ and NGS gene panel analyses became a part of routine clinical practice in 2014^17^, ^18^(Figure 3). The higher peak in the number of diagnosed cases in the year 2015 seen in Figure 3 is attributable to previously‐unsolved cases being re‐analyzed using NGS gene panels and ES, which are now reimbursed by the Estonian Health Insurance Fund. Due to the now widespread use in Estonia of NGS methods in a routine clinical setting, our clinic's findings have contributed to identification of some new IMD, such as an intellectual disability syndrome with single‐nucleotide variants in O‐GlcNAc transferase (OMIM: #300255),^24^, ^25^ a mitochondrial RNA polymerase (*POLRMT*) defect (OMIM: #601778),^26^ a syntaxin‐5 defect (OMIM: #603189),^27^ 3‐methylglutaconic aciduria caused by *CLPB* deficiency (OMIM: #616254),^28^ and a novel (ovario‐) leukodystrophy related to *AARS2* pathogenic variants (OMIM: #612035).^29^ We have also first time detected and contributed in delineation of some rare metabolic defects like atypical presentation of Arts syndrome (OMIM: #301835),^30^ autosomal recessive early‐onset peripheral neuropathy caused by *MCM3AP* gene variants (OMIM: #603294),^31^ *FLAD1*‐associated multiple acyl‐CoA dehydrogenase deficiency (OMIM: **#**255100),^32^ SLC35A2‐CDG defect (OMIM: #314375),^33^ diverse phenotypes of *NDUFB11* gene defect (OMIM: **#**301021),^34^ and early onset mitochondrial disease caused by dominant variants in *SLC25A4* (OMIM: #103220).^35^ In summary, we have identified multiple ways in which use of NGS methods has significantly increased the detection of new and rare IMD.

The overall LB prevalence for IMD found in our study was 41.52 cases per 100 000 LB, which correlates well with previously published European data, in which there were 47.52 cases per 100 000 LB in Europe.^2^ There is no consanguinity, large families, and extensive migration in Estonia, which may influence LB prevalence. Similar nationwide study was recently performed in Austria and a median minimal birth prevalence of 16.9 cases per 100 000 was calculated for the period 1921 to February 2021 (ranging from 0.7 cases per 100 000 in 1921 to 113 cases per 100 000 in 2010).^36^ The visible decline in Figure 2 depicting the LB prevalence of IMD in Estonia over the later years, is due to the fact that, as the study covered the years 1990–2017, children born later in the study period may not yet have been diagnosed (e.g., a person born in 2017 who received their diagnosis in 2018 would not be included in this study).

Comparison of our data to a global meta‐analysis of IMD prevalence^2^ provides some additional insights into the ways in which Estonia may be similar or exceptional in its IMD disease frequencies. Meta‐analysis published in 2018 showed that disorders of amino acid metabolism have the highest LB prevalence of IMD globally with 14.7 cases per 100 000 LB, followed by lysosomal storage disorders with 13.3 cases per 100 000 LB.^2^ Our study found similar results for Estonia, with disorders of amino acid metabolism being the most frequently diagnosed disorder group, while disorders of complex molecule degradation were the next most frequently diagnosed. At the same time, the LB prevalence of complex molecule degradation disorders in Estonia is approximately two times lower than the prevalence suggested in the global the meta‐analysis (7.56 vs. 13.3). The LB prevalence of disorders of amino acid metabolism in Estonia and in the rest of the world are similar (16.82 in Estonia, 14.7 in meta‐analysis). In Europe, the mean LB prevalence of PKU is 12.4 cases per 100 000 LB^37^ and the global birth prevalence of PKU is reported to be 6.6 cases per 100 000 LB.^2^ This indicates that the LB prevalence of PKU in Estonia ‐ 10.96 cases per 100 000 LB – is well‐correlated with the European average and higher than the global average.

On a countrywide comparison basis, we found that the Estonian LB prevalence of MPS of 2.93 cases per 100 000 LB is lower than that of other European countries, including the Czech Republic (3.72),^38^ Germany (3.51),^39^ and Norway (3.08),^40^ but higher that of Poland (1.81),^41^ Denmark (1.77) and Sweden (1.75).^40^ The LB prevalence for all MPS subtypes was estimated in Estonia during earlier period (1985–2006) and was found to be 4.05 per 100 000 LB.^42^ It shows that in case of rare disorders, the prevalence estimation varies in small populations like Estonia. The most common subtypes are MPS II and MPS IIIA, both account for 40% of all MPS. MPS III is also very common combined in other European countries like Poland (48%),^41^ Germany (44.5% MPSIII combined, 31% MPSIIIA)^39^ and Sweden (38% MPSIII combined, 25% MPSIIIA).^40^ Statistical analysis did not show a significant increase in the number of diagnoses per year of disorders of complex molecule degradation in the current study. We interpret this result in light of the distinct clinical features associated with this group, which likely permitted adequate diagnostic power for these disorders throughout the study period, even when the most modern diagnostic methods were not available.

According to the literature, the most common inherited defect of mitochondrial fatty acid oxidation (disorders of fatty acid and ketone body metabolism according to the new classification) is MCAD deficiency, with an estimated global LB prevalence of 5.78 cases per 100 000 LB.^2^ In Estonia, MCAD deficiency frequency was first estimated by testing all newborns for the most common 985A > G variant, and the estimated Estonian prevalence was found to be significantly lower than reported global prevalence ‐ 0.52 per 100 000.^43^ In our present study, we found that the LB prevalence is 0.15 per 100 000 LB in Estonia (only one case diagnosed during the 18‐year study period), compared to 9.9 per 100 000 LB in Germany^44^ and 11.2 per 100 000 LB in Denmark.^45^ The most frequent disorder of fatty acid and ketone body metabolism in Estonia is LCHAD, which according to our study, has a LB prevalence of 0.77 per 100 000 LB. This result is lower than the 1.09 per 100 000 that has been estimated in a previous Estonian study based on carrier frequency.^46^ For comparison, LCHAD has been found to affect 0.71 per 100 000 persons in Germany,^44^ which is very similar to that found in our current study. Our results correlate well with our neighboring countries like Finland^47^ and Latvia.

In conclusion, in spite of having a single center as the only genetic referral center in the country, the small size of Estonia has nevertheless made population‐wide studies feasible. However, the small population also means that sample sizes for rare diseases will likely be small, and therefore, the estimation of prevalence of rare disorders can vary widely in studies with different study periods. Another limitation of our study is that the data from clinics outside the GPMC TUH were only available from the electronic database from years 2008–2017. Statistical analysis has previously shown a significant increase of IMD cases, even without the data from other clinics.^48^ However, disorders like porphyria, which is mainly diagnosed by internal medicine doctors, along with ocular and oculocutaneous albinism, which are diagnosed by ophthalmologists, may be the diagnoses most likely to be absent from GPMC TUH data. Our data analysis showed that 88.9% of the IMD cases were diagnosed in the GPMC TUH or referred to genetic counseling either on suspicion or after confirmation of the diagnosis.

### PATIENT CONSENT

Co‐authors assure that the study was performed according to Helsinki's declaration and in accordance with local protocols and regulations of their institutions. No patient consent was needed.

### ANIMAL RIGHTS

This article does not contain any studies with human or animal subjects performed by any of the authors.

## FUNDING INFORMATION

Katrin Õunap, Karit Reinson, Tiina Kahre, Kai Muru and Elis Tiivoja were supported by Estonian Research Council grant PRG471. Sander Pajusalu received support from the Estonian Research Council grant PSG774, from the European Regional Development Fund, and from the programme Mobilitas Pluss grant MOBTP175.

## CONFLICT OF INTEREST

Elis Tiivoja, Karit Reinson, Kai Muru, Kristi Rähn, Kristina Muhu, Laura Mauring, Tiina Kahre, Sander Pajusalu, Katrin Õunap declare no potential conflicts of interest with respect to the authorship and/or publication of this article.

## ETHICS STATEMENT

The study was approved by Research Ethics Committee of the University of Tartu (278/T‐19 on 19.02.2018 and 288/M‐17 on 17.12.2018).

## Data Availability

All analyzed data consists of patient's personal data and are stored by regulations of the institutions. On request is possible to share anonymized data.
